# Comprehensive evidence-based review on European antitussives

**DOI:** 10.1136/bmjresp-2016-000137

**Published:** 2016-08-05

**Authors:** Alyn Morice, Peter Kardos

**Affiliations:** 1 Head of the Centre for Cardiovascular and Metabolic Research, Hull York Medical School, University of Hull, Castle Hill Hospital, Cottingham, UK; 2 Group Practice and Centre for Allergy, Respiratory and Sleep Medicine at Red Cross Maingau Hospital, Frankfurt, Germany

**Keywords:** Cough/Mechanisms/Pharmacology, Viral infection

## Abstract

Acute cough caused by viral respiratory tract infections is probably the most common illness to afflict mankind. Despite the widespread but ineffective prescribing of antibiotics, there is no specific therapy. Home remedies and over-the-counter medicines are the mainstay for treatment of this short-lived but debilitating condition where cough is a major troublesome symptom. Across Europe, there are large variations in the recommendations made by healthcare professionals for the treatment of acute cough. This has arisen through custom and practice based on the evidence of historical studies performed to standards well short of what would be considered legitimate today. Acute cough is particularly difficult to study in a controlled setting because of the high rate of spontaneous remission and a large placebo effect. Here we detail the validated modern methodology used to assess the efficacy of antitussives and review the drugs commonly used in Europe against these standards.

## Introduction

Acute cough is the most common symptom for which medical advice is sought. It is responsible for over 50% of new patient attendance in primary care and is the major source of consultation in pharmacy practice. Indeed, since symptomatic therapy is the mainstay of management of this generally benign and self-limiting illness, the pharmacist is the key player in the treatment of this condition.

Unfortunately, much of the over-the-counter (OTC) therapy currently recommended throughout Europe is based on custom and practice and is not supported by clinical studies of sufficient quality to meet the standards of modern evidence-based medicine. Here we review the diagnosis and therapeutic options available for the treatment of what is perhaps the most common ailment to afflict mankind.

### Acute cough in common cold and acute bronchitis

A number of overlapping terms are used throughout the world to describe the clinical syndrome of acute viral upper respiratory tract infection (URTI). We suggest that the terminology below really describes different aspects of the same common syndrome.

*The common cold* is defined as an acute viral URTI, with symptoms of sore throat, sneezing, chilliness, nasal discharge, nasal obstruction, cough and malaise.^1^

*Acute cough*, that is a cough arbitrarily defined as being of <2 weeks duration, is one of the most common reasons for patient visits to ambulatory care.^2^

*Acute bronchitis* is a clinical term implying a self-limited inflammation of the large airways of the lung that is characterised by cough without pneumonia, the latter being diagnosed by focal consolidation on examination or on chest X-ray.^3^

It is now recognised that distinguishing between acute cough due to acute bronchitis and/or common cold is not practicable.^4^ ^5^ Only slight pathological differences, if any, exist due to the principal localisation of viruses infecting the respiratory tract. Epidemiological surveys have shown that acute cough in otherwise healthy adults is a self-limiting disease with an average duration of the main symptom, cough of 14 days.^6^ In children, however, acute cough can last an average of 25 days.^7^

Acute bronchitis is caused by viruses (∼50% rhinovirus infection) in at least 90% of cases.^8^ For these infections, no curative (antiviral) treatment exists and antibiotic therapy has been repeatedly shown to be ineffective in patients without pre-existing lung disease.^9^ Despite being a self-limiting disease, acute bronchitis poses both a high symptom burden to individuals and a high financial burden to society, mainly due to work and school absenteeism. Over 50% of new patient consultations to primary care are due to acute cough and up to 85% of cases are erroneously treated with antibiotics—with no impact on recovery.^10^ Apparent success is due to rapid spontaneous recovery and a huge placebo effect.^11^ Unnecessary and uncontrolled use of antibiotics in acute bronchitis contributes to an impending doom of antibiotic resistance.^12^

### Acute cough due to viral respiratory tract infections

In viral respiratory tract infections, sore throat, headache, sneezing, runny nose and nasal congestion appear early in the course of the disease; cough emerges on day 2 or 3 only, but subsequently, from day 4 cough becomes the most bothersome and by far the longest lasting symptom until day 14.^13–15^

Viral infections of the respiratory epithelium cause early release of many inflammatory mediators disrupting the respiratory epithelium, sensitising chemosensitive cough receptors and the neuronal pathway of the cough reflex.^16^ ^17^ Thus, hypersensitivity of the afferent sensory nerves is thought to be the major mechanism causing cough in acute bronchitis, not the production of excessive mucus. Where mild-to-moderate mucus hypersecretion occurs, it is through the superficial goblet cells and submucosal glands.^18^ The incidence of mucus production, if any, seems to be present in common colds in just the first 48–72 hours. An evaluation of the placebo arms (n=774) of several studies in common cold after day 1 show no increase in sputum production.^19^ Thus, in viral respiratory tract infections, sputum expectoration, if any, lasts for a short time and the amount of secretion is small.^20^ From the therapeutic aspect, the treatment of wet and dry cough remains the same and recently a call for the removal of this classification has been made.^21^ Therefore, antitussives with proven efficacy might be the most appropriate treatment to relieve debilitating cough, of whatever character, in acute respiratory tract infections. Worsening bronchial obstruction may only be a risk in patients with pre-existing chronic airway obstruction.^21^

Much of the evidence supporting drug therapy in acute cough is old and of poor quality. There is little randomised controlled trial-based evidence which is of a modern standard. There are also well-known geographical differences in prescribing. For example, in Germany, OTC secretolytics and mucolytics such as ambroxol and *N*-acetylcysteine (NAS) are by far the most popular treatment with a market share as high as 47.4% of the entire common cold OTC market (source: IMS OTC Report). In contrast, in North America, OTC oral decongestant/first-generation (sedating) H1 antihistamines are used most frequently. Both strategies have little supporting evidence. Degrading mucus polymers and lowering mucus viscosity by mucolytic drugs has not been proven effective in treatment of cough in acute bronchitis.^22^ While first-generation antihistamines such as diphenhydramine might be effective in the treatment of cough,^23^ the second-generation ones are not.

### How can we assess the efficacy of antitussive medications?

Since acute bronchitis and acute cough are by definition self-limiting illnesses lasting a few days, it is extremely difficult to distinguish between spontaneous remissions because of the patient getting better naturally from the effect of any medicine which has been administered. Basically, three tools have been used over the years to examine the antitussive activity of the currently marketed drugs. Subjective measures such as the visual analogue scale (VAS) or simply asking the patient whether they think their cough has improved were originally the favoured efficacy measure and many long-established preparations obtained their licence on this basis. Unfortunately, many of the studies are poorly designed with an inadequate number of patients and frequently using a mixed bag of diseases such as chronic bronchitis, tuberculosis and even lung cancer! Clearly, such studies would not be permissible in the modern era. Thus, the evidence base for many traditional antitussive preparations is extremely poor and, in our opinion, would be insufficient to make any claims of antitussive activity in terms of modern ‘evidence-based medicine’.

Two objective methods of assessing cough have been developed. First, in the 1950s, cough challenge was introduced and has been perfected as a highly accurate tool for assessing the cough reflex. The participant inhales an increasing concentration of a protussive substance such as citric acid or capsaicin—the pungent extract of red peppers. The effect of drug on their cough reflex sensitivity is then compared with that of placebo. This methodology is excellent at assessing the characteristics of the study drug, such as its time course, and is frequently used in the development of novel therapies; indeed, it is recommended by the Food and Drug Administration (FDA) as part of the submission portfolio. However, it does not always correlate with subjective measures. For example, morphine has been demonstrated to have a highly effective activity in suppressing cough in some patients, but does not seem to alter cough reflex sensitivity.

The third is a recently developed modality of assessing cough using cough counting.^24^ It has required a number of strides in technical development, particularly in computing power, to establish a reliable methodology using cough counters. Cough counting is now recognised as the ‘gold standard’ for assessing antitussive efficacy by the FDA. Unfortunately, since it is a recently developed technique, very few of the current OTC antitussive medications have been studied using cough counting. Indeed, only a single agent, dextromethorphan, has been demonstrated to be efficacious in this arena.^25^

**Figure 1:**
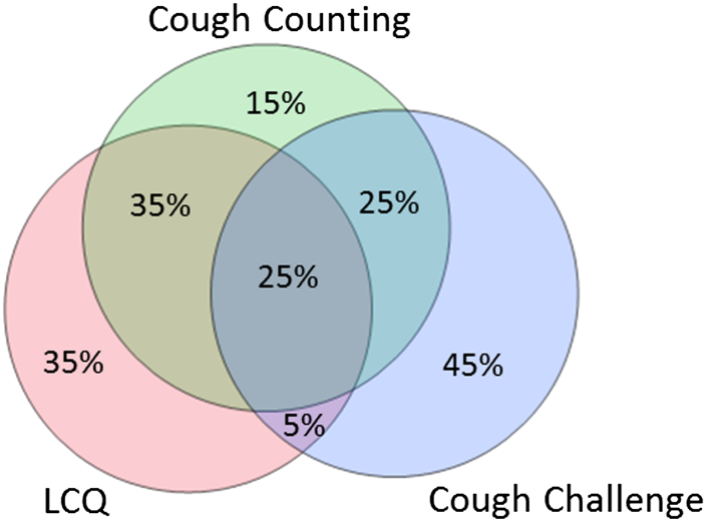
The three methods for studying cough and the relationship between them. LCQ, Leicester cough questionnaire.

It is best to consider the various methodologies for assessing cough as the three overlapping circles of Venn diagram (figure 1). Of the three, subjective measures have proven to be the least reliable and with a few notable exceptions have not been rigorously evaluated. We consider therefore that claims made of antitussive activity solely using subjective criteria provide insufficient evidence of efficacy, a view currently supported by the FDA.

Therefore, in an attempt to promote rational prescribing, we have reviewed the evidence for frequently used treatments in acute cough, particularly from a European perspective. We examined three aspects of drug efficacy on acute cough: the effect on the cough reflex using cough challenge, and both objective (cough recording) and subjective (ie, symptom scores, specific quality of life tools) effects on clinical outcomes.

## The efficacy of antitussive drugs

### Codeine

Codeine is often considered the archetypal antitussive, yet there is little evidence that it has any intrinsic activity of its own. In man, codeine acts as a prodrug, being converted to morphine in the liver by the enzyme cytochrome P450 2D6.^26^ Morphine has been used for centuries in the treatment of cough and indeed has been demonstrated to have efficacy in randomised controlled trials (RCTs). Experience in chronic cough suggests that morphine is only efficacious in about a third to half of patients, others having no effective relief of the symptom. Whether this is also true in the acute bronchitis and cough in common cold is unknown.

Despite its widespread use, there is very little clinical evidence supporting significant antitussive activity for orally administered codeine. In some studies, it has been reported to have no effect on cough challenge or on the sensation of urge to cough, whereas others have reported a small but significant effect.^27^ In two well-designed studies investigating cough due to URTIs, codeine 30 mg, followed by 4 days of dosing four times a day, had no effect greater than placebo syrup, either on an objective initial cough recording or on a subsequent self-reported cough.^28^ In the second study, oral codeine (50 mg) was compared with placebo syrup in 82 participants in a parallel group design using all three measures of cough assessment; again, no effect greater than that of placebo was observed.^29^

The cytochrome system which converts the prodrug codeine to morphine is highly polymorphic.^26^ Some patients are fast metabolisers converting the majority of codeine to morphine at first pass through the liver.^26^ In others, the slow metabolisers, very little codeine is converted. Thus, when prescribing codeine to an individual patient who has not previously used the drug, it is impossible to predict the degree of opiate effects or indeed side effects. Both overdosing or underdosing occurs in an unpredictable fashion. The European Medicines Agency has restricted the use of codeine in children for precisely this reason while the FDA are currently reviewing the use of codeine cough-and-cold medicines in children.^30^ ^31^ Children who are fast metabolisers were observed to have dangerous levels of sedation and suppression of respiration.^26^ We believe that this is not just a problem in the young and that the dangers of codeine far outweigh the limited evidence of efficacy in clinical studies.

### Dextromethorphan

In the domain of cough counting, which is widely regarded as being the gold standard for assessing cough by regulatory authorities such as the FDA, only dextromethorphan has been demonstrated to significantly suppress acute cough using objective measures. In the three studies reported by Parvez *et al*,^27^ 451 patients were observed using acoustic cough monitors. Compared with placebo, there was a highly significant reduction in cough counts with the dose of 30 mg dextromethorphan. To demonstrate true drug effect, dextromethorphan was administered within a capsule form, thus removing the demulcent effect of syrup. This also probably explains the relatively slow onset of action seen in this study. Subsequent studies with dextromethorphan have been performed with the syrup formulation, thus combining the rapid onset of action of demulcent activity with assessed efficacy of the drug. These positive results have been confirmed in a subsequent meta-analysis.^32^

In the second aspect of the assessment of efficacy of antitussive medications, dextromethorphan again passes. There have been multiple studies of the pharmacodynamics and pharmacokinetics of dextromethorphan performed under a variety of cough challenge conditions.^27^ Citric acid challenge is the most common modality, but recently dextromethorphan was found to be superior in the capsaicin challenge model.^33^ Dextromethorphan is revealed to be a drug with a relatively slow onset of action peaking in efficacy after ∼2 hours. Owing to its relatively slow penetration through the blood–brain barrier and consequent retention within the central nervous system, dextromethorphan may have a prolonged antitussive activity, being significantly better than placebo after as long as 24 hours.^34^ Some challenge studies also show that a higher than recommended daily dose of 30 mg may be even more efficacious against cough.

It has proven to be more difficult to obtain subjective evidence for the effect of dextromethorphan in acute cough resulting from common cold infection. As with so much of the literature assessing subjective antitussive effects with a wide range of products, many of the studies are far from rigorous in their execution, using small numbers of participants, often with diverse disease and measuring symptoms without a validated methodology. Perhaps the biggest problem in any subjective measures in cough with common cold is the rapid rate of spontaneous remission in this acute illness, the large placebo effect and the demulcent effect of the syrups. In current preparations containing dextromethorphan, all of these ancillary options are used to enhance activity. Combining the three strands of evidence, it has been estimated that there is an excess antitussive activity due to dextromethorphan at the dose of 30 mg of ∼17%.^32^

### Pentoxyverine

Pentoxyverine citrate is in use as a non-opioid central acting antitussive with very little evidence of clinical efficacy, seen in poorly designed >50 years old clinical studies. Animal studies, however, show efficacy on evoked cough by electrical stimulation or citric acid challenge.^35^ ^36^ In our experience, animal studies are extremely poor at predicting clinical effectiveness of antitussives.

### Butamirate

Butamirate preparations are widely used in Europe as OTC antitussives. Butamirate is thought to have a central mechanism which is neither chemically nor pharmacologically related to that of the opioid alkaloids. Butamirate also possesses non-specific anticholinergic and thus bronchodilator effects. Butamirate is claimed to be effective by the manufacturer in a number of double-blind, randomised, parallel group trials with codeine and other comparators, none of which were placebo controlled.^37–39^ The single placebo-controlled study remains unpublished and is held on file. The effects of butamirate on cough reflex sensitivity, as demonstrated by capsaicin inhalational cough challenge in normal participants, were recently studied in a placebo-controlled six-way randomised cross-over study with dextromethorphan as the positive control. All four doses of butamirate failed to demonstrate greater cough reflex suppression than placebo, whereas dextromethorphan was significantly effective.^33^

### Levodropropizine

Levodropropizine is suggested to be a peripherally acting antitussive which is widely used in southern Europe, particularly Italy. The clinical trials supporting its use in children and adults are summarised in a recent open access meta-analysis.^40^ There were four studies in children and three in adults. Only two studies were placebo comparisons. The paediatric study contained 12 children^41^ with asthma and the adult study (n=40) is not reported in full but is contained in another meta-analysis and appears to have been performed in hospitalised patients, the majority of whom were suffering from chronic bronchitis.^42^ There are thus no placebo-controlled studies demonstrating the efficacy of levodropropizine in acute cough. Of the other comparator studies, only two were in acute cough.^43^ ^44^ By far the largest, and thus contributing most to the results of the meta-analysis, was a non-randomised open observation in children.^43^ All treatments were claimed to be equally effective in reducing subjective measures, but since the comparators have also not been shown to be effective against placebo, little can be made of this claim.

### Ambroxol

Ambroxol is the active metabolite of bromhexine and the most popular drug on the German OTC market (in 2015, 24% of the expectorant market share with an additional 1.7% for bromhexine, source: IMS OTC Report). Most references arise from the 1970s to 1980s and are related to long-term use in obstructive lung disease to prevent exacerbations or are in chronic bronchitis^45^ to ease expectoration. A recent review of ambroxol clinical data^46^ stated that, based on acceptability of study design (ie, randomised, double-blind, controlled) for short-term use in adults, only 3 out of 24 studies were acceptable.^15^ ^47^ ^48^ Only the Matthys *et al*^15^ study investigated acute respiratory tract infection in a large, four parallel arm (some 170 patients in each arm) double-blind quadruple dummy randomised design the effect of 3×30 mg ambroxol days 1–3, 2×30 mg days 4–14, 4×300 mg myrtol (a standardised phytotherapeutic distillate containing 1,18 cineol) 1–14 days and 2×250 mg cefuroxime 1–6 days versus placebo over 2 weeks. Among secondary outcomes were diary data on nightly cough and coughing bouts during the day assessed. All three treatments were similarly effective and significantly better than placebo. The remaining two studies assessed short-term treatment of chronic conditions.^47^ ^48^ Studies in children were conducted only without a control group or versus an active comparator in an open design.

Based on these data, symptomatic efficacy of ambroxol versus placebo on cough is proven in a single RCT.

### N-acetylcysteine

NAC is the second most popular drug for acute cough in Germany with 23.5% of the OTC expectorant market share in 2015 (source: IMS OTC Report), a fact sharply contrasting with the available evidence for this indication. A Cochrane Library meta-analysis of three RCTs with cough at day 7 as the main outcome is available for acute upper and lower respiratory tract infections in a paediatric population.^49^ Statistically significant benefit was seen but the authors felt they were ‘of little clinical relevance’. Another Cochrane review for OTC medications for acute cough in 2014 did not find any references for NAS^50^ nor did a MEDLINE search by the authors of this paper (search terms of N-Acetylcystein AND Cough; N-Acetylcystein AND bronchitis; Acetylcystein AND cough; Acetylcystein AND Bronchitis).

### Oxomemazine

There are no published placebo-controlled, double-blind studies supporting the use of oxomemazine in cough. In a study by Pujet *et al*,^51^ oxomemazine with guaifenesin was compared in a single-blind study with clobutinol in 130 patients with ‘infectious cough’. Cough intensity as assessed by VAS was rapidly reduced in the oxomemazine group, although there was no difference in the overall rate of resolution of cough. In an uncontrolled study in 46 infants under the age of 2,^52^ progress was described as ‘bonne’ in half. Chapuis *et al*^53^ report uncontrolled observations of the ‘novel antihistamine’ on cough with other allergic conditions without any patient details supplied in the manuscript.

### Helicidine

Helicidine is a mucoglycoprotein extracted from the snail *Helix pomatia*. Helicidine has been used for >50 years in France as a cough medicine. In vivo animal studies showed antitussive efficacy in the cats; however, this study was not published.^54^ A placebo-controlled study in adult hospitalised patients with various diagnoses and an observational study in children were also not published.^54^ Studies from the 1950s claim antibacterial effects against *Haemophilus* (now called *Bordetella*) pertussis.^55^ A placebo syrup-controlled study^54^ with objective overnight cough counting in the sleep laboratory in n=30 patients with chronic obstructive pulmonary disease and objectively documented night-time cough was also performed. For the co-primary outcomes cough frequency and cough duration, an almost 50% higher reduction was demonstrated, while for secondary subjective outcomes no significant statistical difference was reported. Thus, there is no published clinical evidence to support helicidine's action in acute cough or acute bronchitis.

### Menthol

Menthol is monoterpene produced by the peppermint plant *Mentha arvensis* from which most of the naturally occurring peppermint oil is extracted. Menthol's cooling activity is through the specific ‘cold’ receptor TRPM8, a member of the transient receptor potential family of nociceptors.^56^ It is primarily located on afferent sensory neurons and is anti-irritant by blockade of voltage-gated sodium channels.

Menthol has an ancient history and has become a stock ingredient of many OTC preparations. Antitussive activity was commercialised by the development of a topical rub by Lunsford Richardson in 1890,^57^ and recent evidence indicates that the antitussive activity of menthol may reside in the activation of nasal as opposed to lung sensory afferents.^58^

Clinical evidence of menthol's activity is sparse with few clinical studies performed to modern standards. Challenge studies in normal participants produce a short-lived reduction in evoked cough. In a small and poorly controlled study, menthol vapour produced a decrease in capsaicin-induced cough.^59^ Cough induced by inhalation of citric acid was reduced in adults by inhalation of menthol vapour compared with air and pine oil control^60^ and in children compared with baseline challenge, but failed to reach significance when compared with placebo.^61^ Surprisingly, there appears to be no published clinical studies on the effect of menthol or of the many products containing it in acute cough or bronchitis.

### Diphenhydramine

Diphenhydramine is a first-generation H1 antihistamine approved in some countries as an OTC antitussive, including the USA and the UK. In Germany, diphenhydramine 50 mg is approved as a hypnotic or antiemetic only. First-generation antitussives in combination with oral decongestants are recommended by the American College of Chest Physicians Evidence Based Guidelines for the treatment of cough in common cold and in the so-called upper airway cough syndrome.^62^ However, despite the title of those guidelines, this recommendation is based on expert opinion.^63^ In cough challenge studies in healthy participants^64^ and patients with acute viral respiratory infection (diphenhydramine combination syrup with decongestant) in adults efficacy could have been established.^65^ However, no symptom or objective cough monitoring-based studies are available for acute cough. There is a clear-cut discrepancy between evidence of efficacy and broad clinical use of diphenhydramine/decongestant combinations for acute cough—despite an important sedative effect (dizziness)—especially in the USA. Table 1 evaluates how European antitussives match up to the modern metrics in cough research.

**Table 1:** RCT-proven efficacy of antitussives by three different cough measurement methods in acute bronchitis

RCT-evidence for drug efficacy	Subjective clinical symptoms	Objective cough recording	Cough challenge	Remarks
Codeine	−	−	−	No convincing evidence of efficacy
Dextromethorphan	+	+	++	Well characterised in objective studies
Pentoxyverine	−	−	−	Only animal studies via 3 clinical studies >50 years old
Butamirate	−	−	−	No placebo-controlled study published
Levodropropizine	+	−	+	6 short-term placebo or active comparator controlled studies n=174
Ambroxol	+	−	−	Many additional non-interventional studies
*N*-acetylcysteine	Children + Adults −	−	−	Many studies for COPD, chronic cough, antioxidant properties
Oxomemazine	−	−	−	Only observational studies
Menthol	−	−	+	Widely used. Vapour antitussive via TRPM8
Helicidine	−	−	−	No clinical evidence of efficacy in acute cough
Diphenhydramine	−	−	−	Broad clinical use

COPD, chronic obstructive pulmonary disease; RCT, randomised controlled trial.

### Antitussives in combination cold therapy

A popular strategy to combat the multiple symptoms in acute viral URTI has been to combine active ingredients. Such a strategy is entirely logical when symptoms require different therapeutic approaches. Thus, the addition of paracetamol to an antitussive to deal both with the cough and headache or myalgia makes therapeutic sense. Similarly, the use of a sedating antihistamine for a nocturnal preparation in combination with the antitussive may well give additional benefit and symptom relief. Some combinations on the market are, however, illogical and based on a poor understanding of the pathophysiology. It is becoming increasingly recognised that there is little evidence to support expectorant activity and, indeed, some agents classified as expectorants have been reported to have anti-inflammatory,^66^ antioxidant^67^ or antitussive activity in challenge studies.^68^ Similarly, expectorants, although widely prescribed in combination treatments, may actually work by decreasing the cough reflex hypersensitivity and thus relieving the sensation of mucus hypersecretion. Perhaps the most interesting studies to provide insight into the mode of action of ambroxol are recent investigations into its ability to block voltage-gated sodium channels located on sensory nerves.^69^ Such an activity is likely to underlie the clinically important local anaesthetic properties which support the use of ambroxol in its indication for sore throat. Sodium channel blockade may also explain some of the other activities of this class of agents through the blockade of neurogenic inflammation.

Perhaps the most important consideration when using combination products is the possibility of a drug interaction, and therefore well-conducted safety and efficacy studies are required. Only studies of adequate power should be considered. As an example, Mizoguchi *et al*^70^ studied 432 participants in a placebo-controlled study of a syrup containing 15 mg dextromethorphan hydrobromide, 7.5 mg doxylamine succinate, 600 mg paracetamol and 8 mg ephedrine sulfate. The primary end point (composite of nasal congestion/runny nose/cough/pain relief scores 3 hours postdosing) showed a highly significant beneficial effect in the group given active treatment (p=0.0002). Each individual symptom score also showed statistically significant improvement 3 hours postdosing (p≤0.017). The next morning active treatment continued to show clinically and statistically significant benefits (p≤0.003). Evidence of benefit with the test syrup was also seen in the higher score for overall night-time relief (p<0.0001) and greater satisfaction on sleep (p=0.002). Adverse events were reported at half the frequency in the active treatment group compared with the placebo and there were no reported events >1% in the population. We suggest that only by the use of large well-controlled and well-designed studies such as this can combination products be recommended with any surety.

### Recommended treatment strategy

- URTIs are benign and self-limiting, and therefore patients with milder symptoms can be safely reassured.
- The demulcent effect of a simple linctus/syrup such as a home remedy of honey and lemon may bring a significant reduction in the cough, albeit of a relatively short duration. This strategy should be the first choice, particularly in children.
- In isolated dry or minimally productive cough, dextromethorphan 30–60 mg per day has the best evidence base.
- When other symptoms are also present, a combination product containing adequate amounts of dextromethorphan should be considered.
- A cough persisting longer than 2 weeks requires additional diagnostic evaluation.

## Conclusions

Acute cough is perhaps the most common symptom to afflict mankind. While it is usually a benign and self-limiting illness, the amount of morbidity endured as a consequence of acute viral respiratory tract infection has enormous consequences on humanity. By the use of evidence-based treatment, significant improvement in patient outcomes can be achieved. While there are many gaps in the knowledge of our therapy in acute cough, our improved understanding of the mechanism of cough hypersensitivity brings rational treatment choices a step closer. A greater understanding of how drugs may normalise this aberrant reflex, thus bringing relief and shortening the duration of illness, may be of enormous benefit to the whole of society.
